# A Methodology to Compare Biomechanical Simulations With Clinical Brain Imaging Analysis Utilizing Two Blunt Impact Cases

**DOI:** 10.3389/fbioe.2021.654677

**Published:** 2021-07-01

**Authors:** X. Gary Tan, Venkata Siva Sai Sujith Sajja, Maria M. D’Souza, Raj K. Gupta, Joseph B. Long, Ajay K. Singh, Amit Bagchi

**Affiliations:** ^1^U.S. Naval Research Laboratory, Washington, DC, United States; ^2^Walter Reed Army Institute of Research, Silver Spring, MD, United States; ^3^Institute of Nuclear Medicine and Allied Sciences, New Delhi, India; ^4^U.S. Army Medical Research and Development Command, Fort Detrick, MD, United States; ^5^Life Sciences Directorate, Defence Research and Development Organisation (DRDO), New Delhi, India

**Keywords:** traumatic brain injury (TBI), computational, modeling and prediction, traffic accidents, magnetic resonance imaging (MRI), diffusion weighted imaging (DWI), apparent diffusion coefficient (ADC), injury assessment

## Abstract

According to the US Defense and Veterans Brain Injury Center (DVBIC) and Centers for Disease Control and Prevention (CDC), mild traumatic brain injury (mTBI) is a common form of head injury. Medical imaging data provides clinical insight into tissue damage/injury and injury severity, and helps medical diagnosis. Computational modeling and simulation can predict the biomechanical characteristics of such injury, and are useful for development of protective equipment. Integration of techniques from computational biomechanics with medical data assessment modalities (e.g., magnetic resonance imaging or MRI) has not yet been used to predict injury, support early medical diagnosis, or assess effectiveness of personal protective equipment. This paper presents a methodology to map computational simulations with clinical data for interpreting blunt impact TBI utilizing two clinically different head injury case studies. MRI modalities, such as T1, T2, diffusion-weighted imaging (DWI) and apparent diffusion coefficient (ADC), were used for simulation comparisons. The two clinical cases have been reconstructed using finite element analysis to predict head biomechanics based on medical reports documented by a clinician. The findings are mapped to simulation results using image-based clinical analyses of head impact injuries, and modalities that could capture simulation results have been identified. In case 1, the MRI results showed lesions in the brain with skull indentation, while case 2 had lesions in both coup and contrecoup sides with no skull deformation. Simulation data analyses show that different biomechanical measures and thresholds are needed to explain different blunt impact injury modalities; specifically, strain rate threshold corresponds well with brain injury with skull indentation, while minimum pressure threshold corresponds well with coup–contrecoup injury; and DWI has been found to be the most appropriate modality for MRI data interpretation. As the findings from these two cases are substantiated with additional clinical studies, this methodology can be broadly applied as a tool to support injury assessment in head trauma events and to improve countermeasures (e.g., diagnostics and protective equipment design) to mitigate these injuries.

## Introduction

Traumatic brain injury (TBI) has become a growing health concern worldwide, leading to a wide range of problems from mild memory deficits to persistent vegetative states (Florence et al., 2018). According to the 2014 Centers for Disease Control and Prevention (CDC) statistics, over 2.8 million cases of TBI are diagnosed in the United States alone (CDC, 2020). The majority of cases are often the result of motor vehicle accidents, contact sports, and falls. The Glasgow Coma Scale (GCS), based on visual, verbal, and motor response, has been used as the standard diagnostic tool to assess head injuries, which are categorized as mild (GCS score: 13–15), moderate (GCS score: 9–12), and severe (GCS score: 8 or less). Depending upon the extent of the insult and the intensity of the impact, injuries can result in combinations of skull fractures, brain hemorrhage, contusions, subdural hematoma, and diffusive axonal injuries, which can be diagnosed from mild to severe range depending on GCS scores (Post et al., 2015). GCS score based diagnosis can be compromised by clinician-to-clinician variability despite attempts to adhere to the guidelines of the American College of Emergency Physicians criteria (Holdgate et al., 2006). Categorically similar GCS scores can be assigned to vastly different brain injuries ranging from localized lesions with/without skull fractures to coup and contrecoup without any presentation of outward signs of injury. Further variation in diagnoses stems from differences in clinical training, available clinical tools, and also the country of training. Despite the engineering perception that bigger impacts worsen the condition, it was found that skull fractures have not consistently correlated with worsened clinical prognosis. Some TBI patients with skull fracture may have mild symptoms from brain injury, while others with no skull fractures can have sustained severe brain injury (Goldsmith, 2001). While moderate to severe injuries are frequently and easily diagnosed based upon impairments that are clinically obvious, mild injuries have been much harder to diagnose, and distinguishing the extent of the injury based upon clinical presentation with GCS scores is typically challenging.

Neuroimaging tools, such as computed tomography (CT) and magnetic resonance imaging (MRI), play a critical role in clinical evaluation of patients with TBI, specifically with mild TBI, and help characterize the types of injury in addition to GCS scores. Acute TBI could lead to both extra-axial and intra-axial injuries. The former manifests as extradural, subdural, and sub-arachnoid lesions. The latter is seen as contusions and parenchymal hematomas, diffuse axonal injury, and vascular injury (Kazam and Tsiouris, 2015). In contrast to CT, MRI can depict the exquisite details of brain parenchymal changes in mild TBI (Wu et al., 2016). Subtleties in some cases of mild TBI with various macrostructural and microstructural changes, including disrupted neuronal tracts and blood-related products in the brain, have been revealed with novel and unconventional magnetic resonance (MR) techniques including three-dimensional MRI, susceptibility-weighted imaging (SWI), echo-planar imaging-based diffusion-weighted imaging (DWI) and apparent diffusion coefficient (ADC) maps, and higher-order diffusion imaging (Van Boven et al., 2009; Hunter et al., 2012; Lu et al., 2014). However, these modalities are infrequently used in the clinical setting due to either lack of high-resolution MR capabilities or a limited number of specialized personnel required to process the data. One of the goals of this study is to understand if these imaging modalities can help provide information for injury prediction from computational biomechanics and correspondence.

Over the years, numerous studies on impact head trauma have been conducted to understand biological and biomechanical mechanisms of traumatic brain injury. Translational and rotational forces from rapid acceleration/deceleration of head (coup and contrecoup injury), blows to the head/falls (local lesions), and combinations of these loading conditions have been shown to cause TBI. Pre-clinical models have been a great resource to understand controlled intracranial injury outcomes, but bridging the gap between the species to translate the data into humans has been a challenge (Cernak et al., 2017). Moreover, the biomechanical parameters that correspond with injury are currently unclear as also are the intracranial manifestations of the insult. Post-mortem human subjects have provided important biomechanical data (Hardy et al., 2007), but the introduction of sensors and other instrumentation alters the tissue response. The data collected in the laboratory setting (e.g., cadaveric studies or controlled experimental impact loading) often do not translate to “real-world” scenario due to the variety of actual loading conditions and the clinical presentation. Computational biomechanics provides an alternative approach to sidestep these confounding issues (Ji et al., 2014). The validated head model, such as in Refs. (Mao et al., 2013; Giudice et al., 2019; Zhou et al., 2019; Budday et al., 2020; Li et al., 2020), can establish full relationships between the impact loading on the head and the internal biomechanical response for actual loading conditions that may not be practical to recreate experimentally. The severity of injury from these biomechanical measures, such as pressures, strains, stress, and their time variances, can be represented using established injury criteria (Zhang et al., 2003, 2004; Fernandes and Alves de Sousa, 2015) and applying such criteria for each element of the model.

Our group has developed a multi-fidelity computational model to represent the dynamics of a pedestrian fall and subsequent structural response of the human head due to an impact (Tan et al., 2020). The developed high-fidelity finite element (FE) model accurately reproduces the complex internal and external structures of the head (Cotton et al., 2015) and has been validated using data from human cadaver tests (Saunders et al., 2018; Tan et al., 2020). This hybrid (biodynamics and biomechanics analyses) approach has simplified the simulation and provided the effect of human body kinematics in calculating stress and strain distributions in the brain. The biomechanical results have been compared with MRI readouts from a case of a motor vehicle accident involving a fall to show the qualitative correspondence between the biomechanical outcomes and the assessed injury (Tan et al., 2020).

A key question from qualitative assessments in our previous research (Tan et al., 2020) was if the clinical data from MR images and their analyses can be quantitatively compared with biomechanical simulation results of real-world blunt impact incidents. The application of different MRI modalities to cross-correlate clinical injury assessment with biomechanical simulations of reconstructing an incident is a missing link. In this work, we aim to develop a method that can map the clinical findings onto quantitative biomechanical results, and create a link between the clinical and biomechanics research on brain injury models and mechanisms due to blunt impact. We analyze two clinical cases of blunt impact, one with coup injury and skull indentation, and the other with coup–contrecoup injury and no skull indentation. The head finite element (FE) model is used to reconstruct the high-fidelity biomechanical response of the skull and brain to blunt impact in a fall based on eyewitness reports and clinical assessment. Different mechanical measures such as pressure, shear stress, principal strain, strain rate, product of strain and strain rate, and strain energy are analyzed to identify possible correspondence between their field values and *in vivo* MRI data. The thresholds of such biomechanical measures for injury severity are determined based on the neuroimaging modality that quantifies the injury. We found that an unconventional MRI modality, such as DWI, is a useful diagnostic tool that can inform computational biomechanics, irrespective of injury type. A methodological implementation of this approach to map computational biomechanical simulation results with clinical image-based data is discussed.

## Methods

### Ethics of Data Collection

A written informed consent was obtained from the individuals for the publication of any potentially identifiable images or data included in this article. The institutional Ethics Committee for the Institute of Nuclear Medicine and Allied Sciences (INMAS) approved the study.

### Clinical Data Collection

Two clinical cases of blunt impact were considered. Both cases had sustained blunt injury to the cranium, but through different mechanisms. While one individual had a motor vehicle accident, the other sustained a fall from a height. Both suffered from a concussion with transient loss of consciousness, corresponding to mild traumatic brain injury.

Imaging modalities like CT and MRI are widely used to assess the extent of damage to the skull and brain parenchyma. Among these modalities, MRI is acclaimed as the most sensitive to detect and delineate brain injury (Amyot et al., 2015). MRI data was collected on a Siemens Skyra 3.0T scanner using a 20-channel phased array head coil and a 45 mT/m actively shielded gradient system. The MR protocol used in this study consisted of the following:

1. Three-plane localizer imaging with TR = 8.6 ms and TE = 4.0 ms;
2. T1 weighted axial images with TR = 2,110 ms, TE = 12.0 ms, TI = 898 ms, slice thickness = 5 mm, field of view = 179 × 220 mm;
3. T2 weighted axial images with TR = 6,000 ms, TE = 100 ms, slice thickness = 5 mm, field of view = 179 × 220 mm, image matrix = 175 × 320;
4. Fluid attenuation inversion recovery (FLAIR) axial images with TI = 2,500 ms, TR = 9,000 ms, TE = 81.0 ms, field of view = 172 × 220 mm, slice thickness = 5 mm, image matrix = 175 × 320;
5. FLAIR coronal images with TI = 2,500 ms, TR = 9,000 ms, TE = 81.0 ms, field of view = 172 × 220 mm, slice thickness = 4.5 mm, image matrix = 175 × 320;
6. T2 weighted sagittal images with TR = 4,550 ms, TE = 87 ms, slice thickness = 4 mm, field of view = 220 × 220 mm, image matrix = 285 × 384;
7. Magnetization-prepared rapid acquisition gradient echo (MPRAGE) with 160 sagittal slices, slice thickness = 0.9 mm, field of view = 240 mm, TR = 1,900 ms, TE = 2.49 ms;
8. Susceptibility-weighted imaging (SWI) with TR = 28 ms, TE = 20 ms and 3D MPRAGE images using TI = 900 ms, TR = 1,900 ms, TE = 2.4 ms, slice thickness = 0.9 mm, field of view = 240 × 240 mm, and image matrix = 218 × 256;
9. Echo-planar imaging (EPI) sequence for generation of DWI and subsequent ADC maps with TR = 8,800 ms, TE = 95 ms, slice thickness = 3 mm, field of view = 230 × 230 mm, and image matrix = 128 × 128.

Each of the above sequences highlights a particular aspect of the tissue, and the integrated use of these sequences provides a holistic understanding of the organ being imaged as well as its underlying pathology. T1-weighted sequences are best for producing the most “anatomical” representation, resulting in images that most closely approximate the macroscopic anatomy of tissues (Mangrum et al., 2012). T2-weighted images are excellent for highlighting pathology as they are very sensitive to changes in water content (Mangrum et al., 2012). FLAIR images are especially useful in the brain, and enable detection of parenchymal edema without the glaring high signal from cerebrospinal fluid (CSF) (Vaswani et al., 2014). SWI images, with their superior sensitivity for paramagnetic deoxygenated blood products enable the detection of hemorrhagic foci in the parenchyma (Tong et al., 2008). Some of the abovementioned sequences are acquired in all three planes of axial, coronal, and sagittal so as to better visualize the details. The MPRAGE sequence is a three-dimensional T1-weighted sequence, which provides a whole brain coverage in a short scan time with isotropic images that can be viewed in multiple planes (Nelson et al., 2008).

DWI exploits the random motion of water molecules in tissue and thereby gives insight into cellularity, cell swelling, and edema (Hagmann et al., 2006). This random motion can be quantitatively assessed using the ADC value, which is displayed as a parametric map that reflects the degree of diffusion of water molecules through different tissues. The normal ADC value ranges from 700 to 1,000 × 10^–6^ mm^2^/s for gray matter, from 670 to 800 × 10^–6^ mm^2^/s for white matter, and from 3,000 to 4,000 × 10^–6^ mm^2^/s for CSF (Sener, 2001). Injury to any part of the brain would alter the ADC value, thus providing an objective quantitative assessment of injury severity. Currently available evidence suggests the ADC change is more sensitive in the detection of sustained brain tissue injury than other available imaging modalities and is better correlated with symptoms (Goetz et al., 2004; Moen et al., 2014).

ADC image data were extracted from 45 transverse slices, 4 mm apart, using ImageJ (Schneider et al., 2012) software, and the data results were saved for further analysis. For 3D reconstruction, the ADC data were mapped to a rectangular box with a 128 × 128 × 45 grid. The resulting ADC model can be viewed in any post-processing application, such as Paraview (Ahrens et al., 2005), and used to compare with biomechanical measures such as pressure, shear stress, strain rate, product of strain and strain rate, and strain energy for the correlation.

### Biomechanics Modeling and Simulation

A multi-fidelity, two-step modeling approach has been used for simulating the brain injury biomechanics in the reconstructed accident. The first step simulates the biodynamics of a whole-body model by the external loading on the body, representing a pedestrian fall. The second step applies the initial condition and effective boundary condition from the biodynamic simulation to the head to determine stresses and strains in the head and brain using FE-based biomechanics analysis. The explicit finite element solver CoBi-FEM (Tan et al., 2017) was chosen for such impact analysis. The solver has been verified in many biomechanics-related projects (Teferra et al., 2018; Saunders et al., 2019; Tan et al., 2020; Tan and Matic, 2020).

To simulate the kinematics of pedestrian fall, an articulated human biodynamic model developed by Tan and Przekwas (2011) was used. This articulated human body model is partitioned into 16 major body components, such as head, neck, chest, and abdomen. Fifteen joints connect these body components to represent the pedestrian. The fast-running biodynamics simulation provides the proper initial and loading conditions for the high-fidelity head FE model, including translational and rotational velocities of the head, and forces and moments at the base of the neck before the head impact. The details of the biodynamic modeling of an articulated human body fall can be found in Tan and Przekwas (2011).

To simulate the biomechanics of blunt impact to the pedestrian due to the fall, the US Naval Research Laboratory high-fidelity human head FE model, developed based upon high-resolution MRI scans of 50th percentile adult male, was used. This model uses 4.5 million tetrahedral elements to discretize the complicated head geometry that includes 29 anatomic tissue components (Cotton et al., 2015). The average characteristic element size is less than 2 mm, which is suitable for capturing impact-induced stress wave propagation in the head. The material properties of the tissue components in the head are based on literature findings (Brewick et al., 2017) that are used to calibrate the material models. The gray matter and white matter are modeled as hyper-viscoelastic materials. The cortical and cancellous bones are modeled as elastoplastic materials to account for large permanent deformation at the impact region. The CSF in ventricles and subarachnoid space surrounding the brain and the spinal cord is modeled as a hyperelastic material with the same speed of sound as water and a very low shear modulus. Average nodal pressure (ANP) linear tetrahedral elements (Bonet et al., 2001) are used to circumvent the locking problem associated with the nearly incompressible biological tissues. Furthermore, this element does not have hourglass instability issue and, thus, provides robust performance in modeling of the large localized deformation in the head (Tan et al., 2020). We have validated the current head model with the experimental results of a cadaveric head (Nahum et al., 1977; Trosseille et al., 1992; Hardy et al., 2007) in both the intracranial pressure (ICP) and brain motion (Saunders et al., 2018; Tan et al., 2020). To produce a typical 20 ms of response time, the simulation required 24 h of computational time using 96 cores on a high-performance computing (HPC) cluster. The cluster is an HPE SGI 8600 system with 48 cores per node, where each node has 192 GB of shared memory, and each core includes an Intel Xeon Platinum 8168/2.7 GHz processor.

From the FE simulation, we obtain various biomechanical variables including the indentation of skull and brain, pressure, principal strain, effective strain, shear stress, von Mises stress, strain rate, product of strain and strain rate, dilatational strain energy density, and distortional strain energy density as listed in Table 1. The strains are based on the Green–Lagrangian strain tensor ***E*** since the simulations involve large strain. The stresses are based on the Cauchy stress tensor. The strain rate is the symmetrized velocity gradient. The effective strain is defined as $\sqrt{\frac{3}{2}\,{\text{E}}^{d}:{\text{E}}^{d}}$ where ***E***^*d*^ is the deviatoric strain of ***E***. Similarly, we can define the von Mises stress and the effective strain rate. The internal energy can be split into the dilatational energy and distortional energy. The dilatational energy is the integration of the product of pressure and local volume change over the entire volume, while the distortional energy is the integration of the product of deviatoric stress and deviatoric strain tensors over the volume. Their maximum or minimum values in the simulation are used to identify the possible correspondence with the *in vivo* MRI injury data. For the 3D spatial dataset, different cross sections of the head are used for both the qualitative and quantitative comparison between MRI data and simulation results. More details on how to make the comparison specifically for individual cases will be described in the results.

**TABLE 1:** Biomechanical variables used to compare with clinical data.

Biomechanical measures	Variables
Deformation	Indentation of skull and brain
Strain related	First principal strain, effective strain
	First principal strain rate, effective strain rate, shear strain rate
	Product of effective strain and effective strain rate
Stress	Pressure, maximum shear stress, von Mises stress
Energy	Dilatational strain energy density, distortional strain energy density

## Results

### Clinical Interpretation of Brain Injury Due to Fall

**FIGURE 1:**
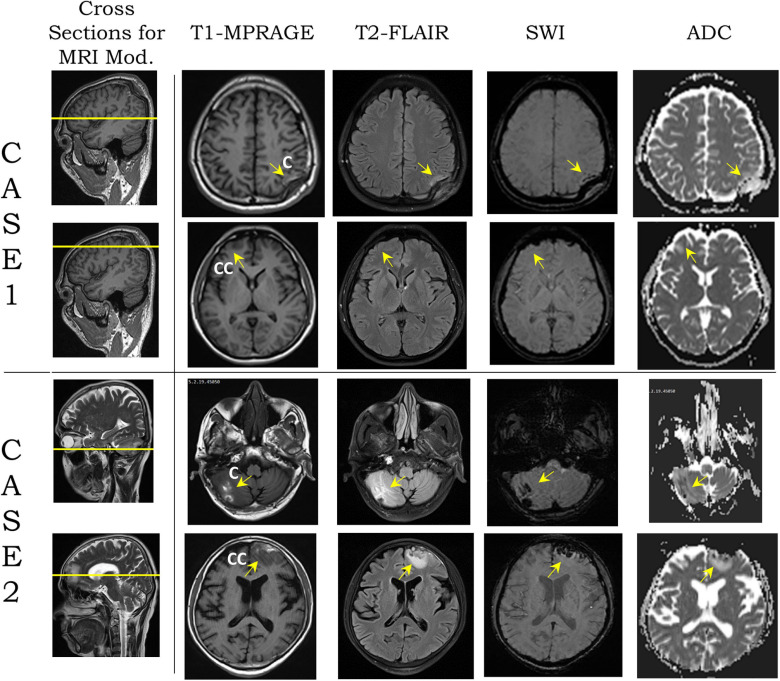
Modalities [T1-magnetization-prepared rapid acquisition gradient echo (MPRAGE), T2-fluid attenuation inversion recovery (FLAIR), susceptibility-weighted imaging (SWI), and apparent diffusion coefficient (ADC)] from magnetic resonance imaging (MRI) images on two transverse planes, shown by yellow lines on sagittal plane section for cases 1 and 2 (second column from left). For each case, the top row shows the coup region images from blunt impact, and the bottom row shows the contrecoup region. Yellow arrows show the coup and contrecoup on modalities. C and CC stand for coup and contrecoup regions, respectively.

Table 2 shows the two cases used in this study. High-resolution image data of case 1 was obtained from an MRI scan of a young South Asian adult, who had sustained head injury from a road traffic accident, a few days prior to the scan. The hospital report indicated that the individual was a pedestrian who was hit by a speeding motor vehicle from the front, sustained a concussion due to the backward fall on the ground, and suffered from loss of consciousness for a period of approximately 20 min. The GCS score at the time of hospital admission, in less than 1 h from the blunt impact, was 13, which is attributed to a mild brain injury. A depressed fracture of the skull with underlying hemorrhagic changes was observed in CT imaging. The MRI data revealed a depressed fracture of the right parietal bone of the skull with a hemorrhagic contusion in the underlying brain parenchyma and associated subdural hematoma in the first row of Figure 1. The maximum skull indentation at the impacted region measured from the MRI image is found to be approximately 9 mm. No contrecoup injury was observed in the actual medical images (second row of Figure 1).

**TABLE 2:** Clinical assessment of brain injury due to fall accidents.

Case number	Cause of blunt impact	GCS score at emergency room	Injury assessment
1	Road traffic accident; backward fall	13	Skull indentation; coup injury
2	Fall from height; occipital collision	15	Coup and contrecoup injury

Case 2 was a middle-aged South Asian adult, who accidentally fell to the ground from a height, according to the hospital report. The individual hit his head in the occipital region and sustained a head injury with a transient loss of consciousness for approximately 15 min. At the time of admission to the hospital, the patient had regained consciousness and had a GCS score of 15. CT scan revealed hemorrhagic contusions in the right cerebellar and left frontal regions, but no skull fracture or visible skull deformation was observed. He underwent an MRI scan several days after injury. This, too, revealed hemorrhagic contusions in the right cerebellar and left frontal regions with a fine extra-axial subdural fluid collection in the left fronto-temporo-parietal regions suggestive of a coup–contrecoup injury (last two rows of Figure 1). The complete set of FLAIR images for both cases can be found in Supplementary Figures 1, 2 which show the gross injury patterns well.

### Correspondence Between Clinical Images and Biomechanics

#### Conditions of Biomechanical Simulations

**FIGURE 2:**
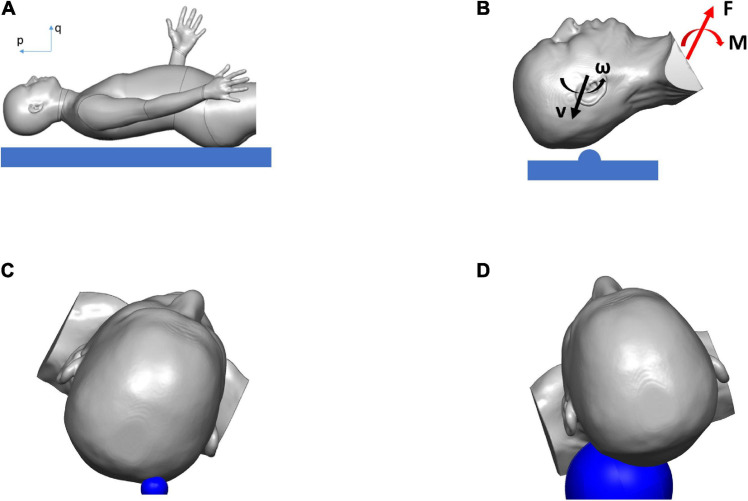
Simulation results showing the biodynamics of the fall, resulting in blunt impact to the head: **(A)** head position just before hitting the ground, **(B)** head with initial velocities hitting hemispherical object and rotating about T1, and producing the forces and the moments applied to the head and neck, **(C)** head hitting hemispherical object of radius 15 mm (in blue) in case 1, **(D)** head hitting hemispherical object of radius 60 mm (in blue) in case 2.

The biodynamics of the fall was modeled using the case reports documented by the hospital, as well as clinicians’ assessment of the most likely trajectory of the fall and the impact (Figure 2A). For the cases simulated here, the assumptions were: (i) The head was rotating backward around the first thoracic vertebra (T1) before hitting the ground (Figure 2B). (ii) The head impacted a solid hemispherical object on the ground. The distance between T1 and the center of the mass of the head was approximately 0.2 m. For case 1, the biodynamic simulation of the fall suggested a 30 rad/s angular velocity of the head around joint T1 when impacting a 15-mm radius solid object (Figure 2C) to generate a comparable skull indentation as in the MRI image. For case 2, the simulation suggested a 15 rad/s angular velocity of the head around joint T1 when impacting a 60-mm radius solid object (Figure 2D) without creating a visible skull deformation at the impact region. The size of the solid object for each case was determined based on the information from the emergency room (ER) documentation and clinical report. The forces on the head due to the impact were the boundary conditions used to simulate the biomechanics of the blunt impact to the head. Both simulations were carried out for a total duration of 20 ms from the point of contact between the head and the hemispherical object.

#### Kinetics of Simulated Blunt Impacts

**FIGURE 3:**
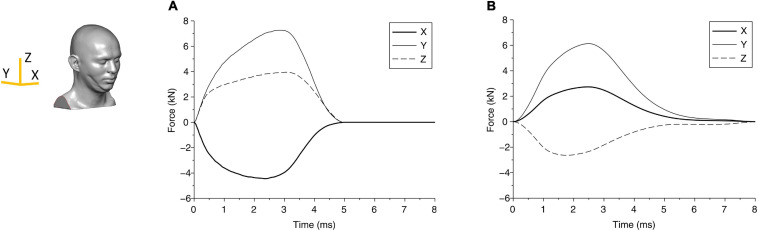
The force components due to the blunt impact between the head and the impactor are shown for **(A)** case 1 and **(B)** case 2. The axes are shown in the head finite element (FE) model.

**FIGURE 4:**
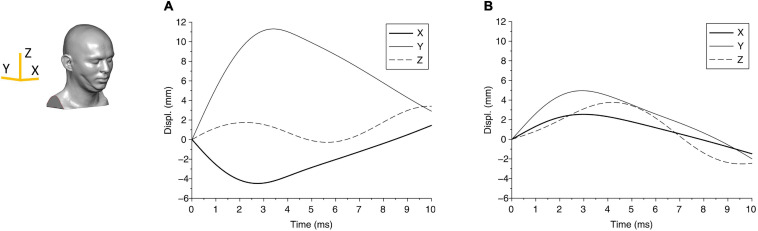
The displacement components of the center of gravity of the head are shown for **(A)** case 1 and **(B)** case 2. The axes are shown in the head FE model.

From the biomechanical simulations, the time histories of contact force components for case 1 and case 2 are shown in Figure 3. The force components rise faster and last a much shorter duration (approximately 5 ms) in case 1 compared with that in case 2 (time for the force to return to zero is approximately 8 ms). The displacements of the head at the center of gravity are shown in Figure 4, which show that the head initially moves toward the impactor and then rebounds around 2.5 ms for both cases.

**FIGURE 5:**
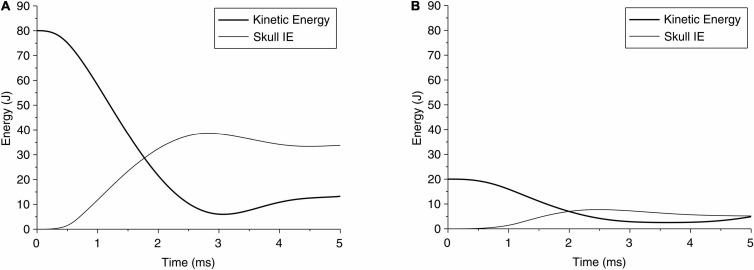
Time history of kinetic energy of the head during impact of head due to a fall, and the internal energy (IE) of skull absorbed by the skull for **(A)** case 1 and **(B)** case 2.

The change in kinetic energy from the impact of the pedestrian’s head with the solid object is shown in Figure 5A for case 1 and Figure 5B for case 2. Figures 5A,B also show the energy absorbed by the skull due to elastic and/or plastic deformation during the impact. The kinetic energy is maximum at the onset of the impact, at approximately 80 J. Over time, i.e., during the contact, this energy is partly absorbed by the skull to cause elastic and plastic (permanent) deformation of the skull, and partly transmitted into the brain. Figure 5A shows that for case 1, the maximum energy absorbed by the skull is approximately 50 J, which causes skull indentation (Figure 3A). This energy is responsible for large local strains and pressure build-up in the coup region of the brain in case 1. The concentration of the strains in the brain at the point of impact perhaps reflects the higher energy being absorbed by the brain in this region. The change in energy distribution for case 2, shown in Figure 5B, on the other hand, shows a different pattern. The total kinetic energy due to the impact is approximately 20 J, but a smaller proportion of this energy (approximately 8 J) is absorbed by the skull, and most of it, we assume, contributes to an elastic deformation of the skull. The energy transmitted into the brain is distributed more evenly in the coup region, causing the coup–contrecoup phenomenon in the brain.

**FIGURE 6:**
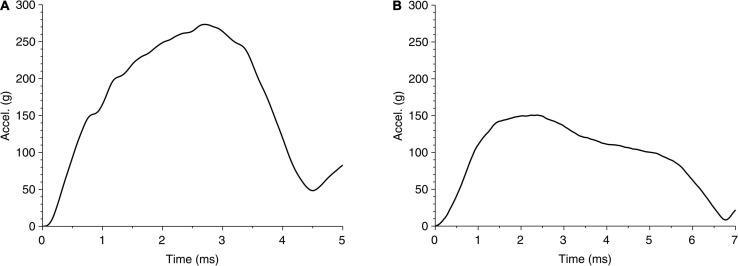
Time history of head acceleration during impact of head due to fall for **(A)** case 1 and **(B)** case 2.

The global response in cases 1 and 2 can also be represented through head accelerations over time, shown in Figure 6. Although both acceleration profiles are similar, case 1 has a higher peak (270 g) than case 2 (150 g); on the other hand, case 1 has a shorter period of contact (approximately 4.5 ms) than case 2 (approximately 6.7 ms). These differences suggest that both the impact energy and impactor can affect the head acceleration. While the acceleration profile can suggest a measurable parameter, the limited data set here is not adequate to relate acceleration to any of the region-specific clinically identified brain injuries for these cases. For reference, the US Army aircrew helmet requirements use an average peak acceleration of 150 g with a maximum of 300 g, for the blunt impact pass-fail criteria (McEntire and Whitley, 2005).

#### Computational Biomechanical Outcomes

**FIGURE 7:**
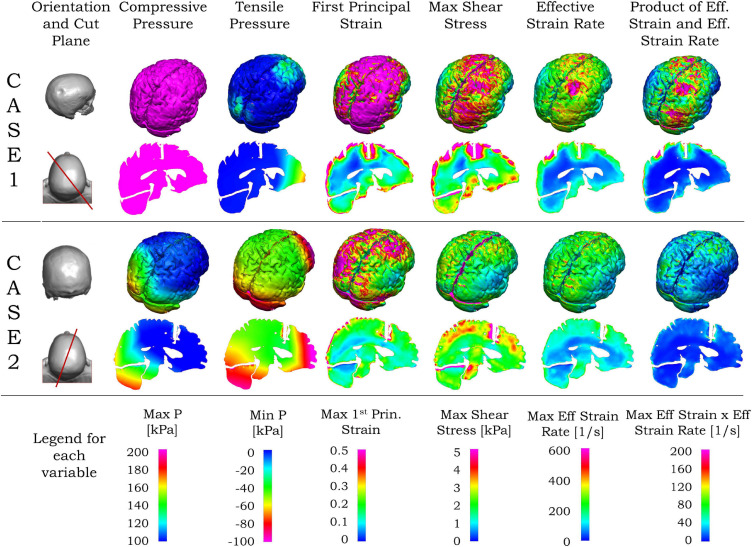
Simulated blunt impact biomechanical responses for cases 1 and 2. For both cases, the second column from left shows the head (top), and vertical plane through the coup–contrecoup line (bottom). For each case, the upper row shows the biomechanical contours for the brain, and the bottom row the contours on the vertical plane passing through the coup–contrecoup line. The last row shows the legends for the maximum of each biomechanical variable specified on the top row. Only data for cerebrum, cerebellum, and brainstem regions are shown.

In the following paragraph, we present the results related to the observed injury in the brain for these two cases. The contour of maximum effective strain rate on the brain during 20 ms of biomechanical simulation for case 1 is shown in the first row of Figure 7. Among the three types of strain rates commonly used, the peak value of the maximum shear strain rate is the largest, the effective strain rate is slightly smaller, and the maximum principal strain rate is about one-half of the maximum shear strain rate or effective strain rate. When comparing the contour pattern of strain rate in the brain, these three strain rates are similar in their respective value ranges in both cases (shown in Supplementary Figure 3). In this paper, we use the effective strain rate for the comparison. Due to the local skull indentation of case 1, the brain is in compression, and the minimum pressure shown in Figure 7 is small. Similar to observations from clinical imaging of case 1, the skull deformation is observed at the impact region with anatomical accuracy. The large value of maximum effective strain rate mainly appears near the indented area, similar to the injury locations seen in Figure 1 and Supplementary Figure 1. In contrast to case 1, the skull in case 2 has no observable deformation, and the strain rate is relatively small and distributed around the brain, as shown in the third row of Figure 7. The contour of minimum pressure on the brain during 20 ms of computational simulation for case 2 is also shown in Figure 7. The minimum pressure around -100 kPa was found at both coup and contrecoup regions, similar to the injury locations as observed in case 2 of Figure 1 and Supplementary Figure 2.

Unlike the effective strain rate, the large values of first principal strain, maximum shear stress, and the product of effective strain and effective strain rate in Figure 7 are seen in the area beyond the indented region in case 1. In case 2, the maximum shear stress, effective strain rate, and the product of effective strain and effective strain rate are relatively small and again distributed around the brain in Figure 7. In both cases, the von Mises stress contours are similar to those of maximum shear stress, while the contours of strain energy density are similar to those of the product of effective strain and effective strain rate and, thus not shown here.

Different cross sections of the 3D head can be used for comparison between MRI data and simulation results. From the MRI images, the most injured regions are near the impact site and/or along the impact direction. Compared with other planar views such as sagittal, transverse and coronal planes, the plane passing through a line joining the impact location and its diametrically opposite point on the skull is more representative for visualization of the injury in the brain. Such a vertical plane, shown by the red lines in the left column of Figure 7, was chosen to represent the transitions in the ADC values from the MRI as well as the simulated biomechanical measures. Details of the computed maximum pressure, minimum pressure, maximum first principal strain, maximum shear stress and maximum effective strain rates for the two cases are elaborated in the second and fourth rows of Figure 7. For case 1 with the coup injury, only the maximum effective strain rate in the second row of Figure 7 shows a large value in the coup region and smaller values in other areas. For case 2, with the coup–contrecoup injury, only the minimum pressure in the fourth row of Figure 7 shows the large value at both coup and contrecoup regions. Other variables do not show correspondence with the clinically identified extent of injury. Note that the maximum pressure is large in the entire brain in case 1, while it is large only at the coup region in case 2. The time to reach the maximum for all biomechanical variables in the brain shown in Figure 7 during the impact events is in Table 3.

**TABLE 3:** The time to reach the maximum of different variables in the brain (ms).

Case number	Compressive pressure	Tensile pressure	First principal strain	Maximum shear stress	Effective strain rate	(Effective strain) × (Effective strain rate)
1	2.9	0.4	4.3	4.1	1.9	4.7
2	15.1	13.7	9.7	13.5	17.1	11.4

#### Comparison of Magnetic Resonance Imaging-Apparent Diffusion Coefficient Data With Computational Biomechanics

**FIGURE 8:**
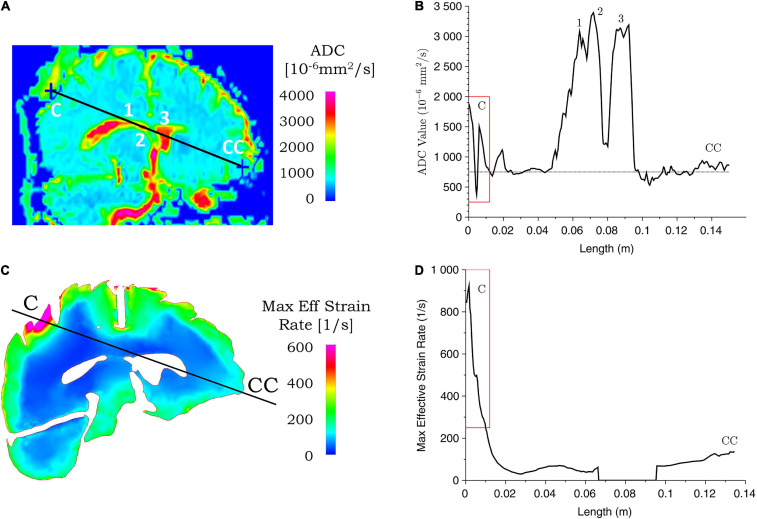
Correspondence between ADC values from clinical data and simulated maximum effective strain rate for case 1. ADC **(A)** and maximum effective strain rate **(C)** contours are shown for the coup–contrecoup plane; ADC **(B)** and maximum effective strain rate **(D)** plot along the coup–contrecoup (C-CC) line with the points 1, 2, and 3 (inside ventricles) marked in **(A)**. The dotted line represents approximate mean value of ADC in **(B)**. Maximum effective strain rate shown in cerebrum, cerebellum, and brainstem only.

**FIGURE 9:**
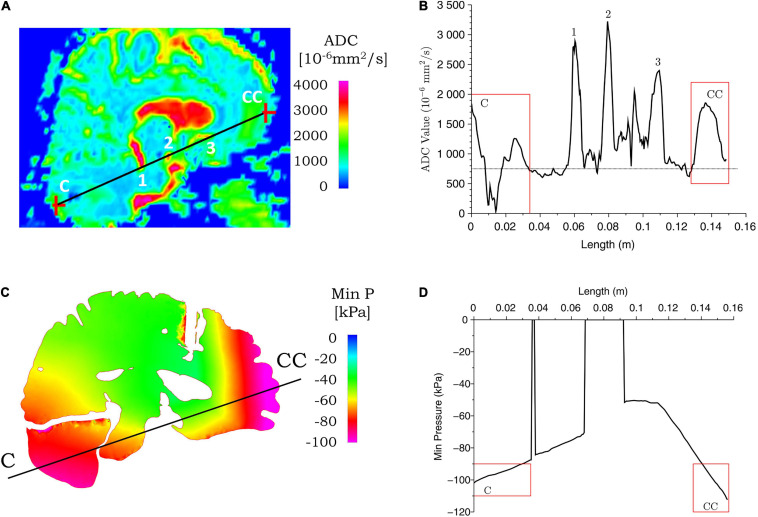
Correspondence between ADC values from clinical data and simulated minimum pressure for case 2. ADC **(A)** and minimum pressure **(C)** contours are shown for the coup–contrecoup plane; ADC **(B)** and minimum pressure **(D)** plot along the coup–contrecoup (C-CC) line with the points 1, 2, and 3 (inside ventricles) marked in **(A)**. The dotted line represented approximate mean value of ADC in **(B)**. Minimum pressure shown in cerebrum, cerebellum, and brainstem only.

The ADC maps for the two cases considered here are compared with the biomechanical measures in Figures 8, 9. We identified the location of the center of impact region of the head from the MRI data, and the point on the diametrically opposite surface of the cranium, and termed them as the coup and contrecoup points, respectively. We compare below, the injury assessment of ADC data with biomechanical measures along the coup–contrecoup line, as a representative way to identify the correspondence between clinical analysis and simulation results.

Figures 8, 9 show the ADC contour maps on the left and the corresponding ADC values along the coup–contrecoup line on the right in the top row, and maximum effective strain rate and minimum pressure contour maps and the corresponding distributions along the same line in the bottom row, respectively, for cases 1 and 2. All ADC values are compared with respect to an approximate mean value of 750 × 10^–6^ mm^2^/s of gray matter and white matter, which is displayed by the dotted lines in Figures 8B, 9B.

For case 1, in the brain injury region (i.e., the coup region), the relative change in ADC from the mean normal value ranges from -450 to 1,150 × 10^–6^ mm^2^/s (Figure 8B), and this significant change is observed up to a depth of approximately 10 mm from the cerebral cortex. In the same region, i.e., at the same depth from the brain cortex, computational simulation shows the maximum effective strain rates to be from 250 to 950 s^–1^. In the contrecoup region, there is no observable change in ADC values from the baseline, while the effective strain rate increases up to 150 s^–1^. This suggests that smaller strain rates may represent correspondingly small or no noticeable ADC changes in injured brain tissue.

For case 2, a comparison of ADC map with simulated minimum pressure at the coup–contrecoup cross section is shown in Figure 9. At the end of the 20-ms contact–rebound simulation period, the large minimum/tensile pressure appears at both the coup and contrecoup regions. However, when compared with the coup region, the contrecoup exhibits larger tensile pressures in the superficial cortical structures. In the coup region of the brain injury, the relative change in ADC shows a clearly identifiable range from -750 to 950 × 10^–6^ mm^2^/s, and this higher value is observed up to a depth of approximately 30 mm from the cerebral cortex. In the contrecoup region of the brain injury, the ADC change increases to 1,100 × 10^–6^ mm^2^/s, and this higher value is observed up to a depth of approximately 20 mm from the cerebral cortex. In the same coup region at the same depth, computational simulation shows minimum pressure from -90 to -100 kPa. In the contrecoup region at the same depth, computational simulation shows a minimum pressure from -90 to -110 kPa. Table 4 summarizes the ADC changes from the mean value in the coup and contrecoup regions and biomechanical parameters and the influence of depth for the two cases. Additional comparison between ADC data and simulation results in the transverse plane showing similar outcomes for the two cases can be found in Supplementary Figures 4, 5.

**TABLE 4:** Comparison between the change in ADC and biomechanical measures for the two cases.

Case number	Range of ADC change and depth in the coup region	Range of ADC change and depth in the contrecoup region	Depth of effective strain rate (>250 s^–1^) in the coup region	Depth of pressure (<-90 kPa) in the coup region	Depth of pressure (<-90 kPa) in the contrecoup region
1	(-450,1,150), ∼10 mm	−	∼10 mm	−	−
2	(-750,950), ∼30 mm	(0,1,100), ∼20 mm	−	∼30 mm	∼20 mm

#### Comparison of Injury Types in Case 1 (Skull Indentation) and Case 2 (Coup and Contrecoup Injury)

At the impact location (i.e., coup region), the maximum ADC change for case 1 is around 1,150 × 10^–6^ mm^2^/s, which is larger than that for case 2 (around 950 × 10^–6^ mm^2^/s). When the deformation of the tissue from brain surface is considered, the depth of ADC change is relatively smaller in case 1 (approximately 10 mm) when compared with case 2 (approximately 30 mm). For case 2, the maximum ADC change in the coup region (around 950 × 10^–6^ mm^2^/s) is smaller compared with that at the contrecoup region (around 1,100 × 10^–6^ mm^2^/s). The region of the brain normal to the interior surface of the skull shows noticeable ADC change at the coup region (approximately 30 mm) compared with that at the contrecoup (approximately 20 mm). In comparison, for case 1, the maximum effective strain rate is above 250 s^–1^ up to a depth of 10 mm in the coup region but less than 150 s^–1^ in the contrecoup region. For case 2, the minimum pressure below -90 kPa is 30 mm into the brain tissue in the coup region and 20 mm in the contrecoup region. In addition, the minimum pressure in the coup region (-100 kPa) is less than that in the contrecoup region (-110 kPa). The similarity of both the ADC change and simulated minimum pressure appears to be that the contrecoup injury is more severe than the coup injury but affects a smaller depth in the brain.

#### Sensitivity of Simulation Results Related to Impact Conditions

Sensitivity analysis was carried out with computational simulations by assessing the effect of impact condition on the injury outcome from the clinical analysis. A number of simulations were performed by changing the angular velocity of the head in case 1 and case 2. As shown in Table 5, for case 1, when the angular velocity is varied between 28 and 32 rad/s, the skull indentation changes from 8.6 to 9.5 mm and the head acceleration from 245 to 290 g. The region of the brain that exceeds the effective strain rate of 250 s^–1^ also varies, in which the depth of this region from the brain outer surface along the coup–contrecoup line changes from 8.8 to 10.2 mm (see Figure 8 for the coup–contrecoup line and the plot of strain rate contour for angular velocity of 30 rad/s). At angular velocities lower than 28 rad/s or higher than 32 rad/s, both the skull indentation and injury depth in brain become increasingly different from those measured from the MRI data. This suggests that an angular velocity close to 30 rad/s reasonably represents the actual accident condition being modeled.

**TABLE 5:** Sensitivity of simulation results for the two cases.

Case number	Impact angular velocity (rad/s)	Head acceleration (g)	Depth of indentation (mm)	Normal depth into brain (mm)	
				Strain rate (>250 s^–1^)	Pressure (<-90 kPa)
1	28	245	8.6	Coup: 8.8	None
	30	270	9.1	Coup: 9.8	None
	32	290	9.5	Coup: 10.2	None
2	12	125	None	None	Coup: 15 Contrecoup: 17
	15	150	None	None	Coup: 30 Contrecoup: 20

In case 2, as shown in Table 5, when the angular velocity is varied between 12 and 15 rad/s, the head acceleration changed from 125 to 150 g with no visible deformation on the skull. The region of the brain with a maximum tensile pressure in the coup and contrecoup regions below -90 kPa also changes. On the coup side, the depth of this region (i.e., with a tensile pressure below -90 kPa) ranges from 15 to 30 mm. On the contrecoup side, the depth of this region is increased from 17 to 20 mm (see Figure 9 for the coup–contrecoup line and the plot of minimum pressure contour for angular velocity of 15 rad/s). With angular velocities smaller than 12 rad/s, little or no injury is seen at the coup or contrecoup regions based on the given pressure criterion. At angular velocities greater than 15 rad/s, at the coup region, the skull deforms plastically, the tensile pressure disappears, and the strain rate gradually increased to over 250 s^–1^. This suggests that the angular velocity of 15 rad/s is a realistic value to be used in the modeling of case 2.

The effect of the impactor size is also examined by using the same impact velocity for these two cases. When an impactor with a large radius of curvature is used, a significantly higher angular velocity is needed to indent the skull to the same depth seen with an impactor with a small radius. For example, if the same angular velocity of 15 rad/s is used, case 1 shows an approximately 3 mm skull indentation and a small focal injury at the coup, while case 2 of relatively large impactor shows no visible skull deformation, but a coup–contrecoup injury is apparent.

## Discussion

### Clinical Observation of Injury Patterns

The present study, based upon MRI findings, has considered two cases from subjects who sustained blunt trauma to the head with characteristically two different types of injury that were diagnosed as mild TBI. Both cases had GCS scores of 13–15 (mild TBI) at the time of diagnosis by the attending physician. Based on eyewitness report and emergency room medical assessment documentation, the way the injuries were sustained due to the falls are different. In the first case, a young adult male sustained a road traffic accident, fell backward, hit an object with a small radius of curvature, and had a depressed fracture of the skull at the site of impact. The underlying brain parenchyma showed evidence of a hemorrhagic contusion. Notably, no contrecoup lesion was visualized as reported in the clinical assessment documentation. Since the underlying hemorrhagic contusion was limited to a site of the injury in the brain parenchyma, adjacent to the depressed skull, certain changes in ADC values were not demonstrated appreciably due to the potential infiltration of blood and meningeal encroachment into the brain parenchyma, but a significant lesion site is visibly identifiable. In addition, magnetic susceptibility artifacts generated by the overlying skull bone could have contributed to those ADC values. No other change was observed in the range of ADC values specific to tissue/region in any other parts of the brain parenchyma.

In the second case, a middle-aged male had an accidental fall from a height, sustained a hemorrhagic contusion in the left cerebellar hemisphere and right frontal lobe corresponding to a coup and contrecoup lesion, respectively, with no skull fractures. The lesions reported in the clinical assessment documentation and seen in the images in this paper were larger than those in the previous case, likely owing to the impact condition and the intact skull during impact. These changes were mirrored in the ADC maps. The hemorrhagic areas showed restricted diffusion corresponding to reduced ADC values at the coup, a well-known phenomenon due to blood at the lesion site (Kang et al., 2001; Shah et al., 2004). These areas were surrounded by edema, which corresponded to an area of increased ADC at both sites (coup and contrecoup). This is owing to the relatively unrestricted diffusion in areas with increased interstitial fluid.

Both of these cases were diagnosed similarly as mild TBI from the GCS scores, but the clinical presentation with skull indentation with a localized lesion in case 1 is significantly different from case 2 with no visible skull fractures but presented with coup–contrecoup lesions when observed through MRI. The nature of the injuries imparts different biomechanical loading on the head, producing characteristically different injury patterns albeit with a similar diagnosis. These injuries in the real-world scenario with mild TBI are not necessarily diagnosed using MRI unless enrolled in a clinical study to understand the intricacies of case-to-case differences. Thus, reconstructing the incident from medical reports can provide objective information on possible real-time injury patterns and diagnosis decisions at the site of injury or in the emergency room.

### Possible Injury Mechanisms and Injury Criteria

Comparing the simulation results and ADC data for case 1, the maximum effective strain rate above 250 s^–1^ is found to correspond with significant ADC changes in the coup side of the brain. High strain and stress values are seen not only in the coup but also in other regions of the brain that do not show observable ADC changes. This suggests that if a stress- or strain-based injury criterion is used to evaluate the brain tissue damage, the threshold for injury cannot be constant and needs to depend inversely on the strain rate. For impact loading at a lower strain rate, the stress or strain threshold is higher, while at a higher strain rate, it become slower. In other words, the brain tissue, like other materials, may become more brittle at a higher strain rate (Yang and Zhang, 2019). This is probably because micromechanically, there is not enough time for dispersion of the linear momentum into other regions of the brain, and the localized deformation is manifested through skull indentation and coup injury. Conversely, *in vitro* tissue experiments showed that for the same strain magnitude, the extent of injury and pathophysiology can be influenced by strain rate (Bar-Kochba et al., 2016) and high water content in the brain tissue (Prabhu et al., 2019).

On the other hand, for case 2, the minimum peak pressure below -90 kPa seems to be the biomechanical parameter that best represents the ADC change associated with the coup–contrecoup injury. There can be several possible mechanisms for coup–contrecoup injury. One possibility is a mismatch between brain and skull motions causing the coup and contrecoup injury. To evaluate this as a possible mechanism, the brain motion relative to the skull was examined for the duration of the simulation, and no direct skull–brain contact was found during the impact. A longer duration simulation may reveal this information, which will be considered in future studies. Another possible mechanism is the stress wave inside the cranium. In the current simulation for this case, we found a stress wave originating and propagating from the impact site on the skull, and being reflected from the free surface from the skull on the contrecoup side that appears to create the tensile/negative pressure in the brain. Since materials like brain tissue are weaker in tension than compression, we conjecture that the excessively large tensile pressure and possibly some cavitation could contribute to brain injury at the contrecoup site as shown in the MRI and ADC data. Experimental and simulation data from impact loading to tissue and gel samples (Kang and Raphael, 2018) showed rapidly increasing tension, which contributed to cavitation and may cause extensive damage to the surrounding tissue. We surmise that while stress wave propagation in the head may be a mechanism for blunt impact injury, current understanding of possible brain cavitation in the brain tissue is limited.

For case 1, the higher kinetic energy seems to localize the effect by indenting the skull and creating large deformation in the coup region in the brain, with little or no effect on the contrecoup side of the brain. We see increased pressure throughout the brain, which may have prevented any clinically identifiable contrecoup injury, perhaps due to an absence of tensile pressure in this region. In case 2, on the contrary, a lower kinetic energy is distributed over a larger area of the skull and the brain, resulting in a characteristic coup–contrecoup effect, which may be attributed to how energy and acceleration change over time (Figures 5, 6). We conjecture that the rapid transition from kinetic energy to internal energy in case 1 compared with the gradual transition in case 2 may explain the different deformation mechanisms in the brain between the two cases. The peak acceleration in case 1 is higher than that in case 2, which corresponds to that of clinically reported GCS score severity. It also appears that flattening the head acceleration curve with a longer duration and lower peak could reduce brain injury severity when comparing the accelerations between case 2 and case 1. This principle based upon the rate of energy and acceleration increments can be applied to study various injury types due to mechanical trauma (e.g., blunt impact and blast exposure) to the brain.

### A Methodology to Map Clinical Assessments With Biomechanical Simulations

Most, if not all, analyses of traumatic brain injury to date have been approached through three major disciplines: pre-clinical, clinical, and computational studies. The pre-clinical studies have been conducted to understand the pathogenesis through molecular, biochemical, imaging, and behavioral studies by replicating real-world injury scenarios using animal subjects. The clinical studies have used Warfighter and civilian clinical cases of TBI with several techniques, such as imaging data and post-mortem histopathology/biochemical analyses. The computational modeling and simulation approaches have attempted to quantify biomechanical responses at multiple scales to predict the observed injury in pre-clinical and clinical scenarios, both with and without protective equipment. All three approaches have explored blast overpressure, blunt, and ballistic impact loadings.

The clinical imaging and computational simulation data for the two cases considered in this paper represent two aspects of injury presentation. The key clinical aspect here was the utilization of post-incident T2-FLAIR to establish the type and location of injury, and ADC maps derived from DWI modality to obtain quantitative data for clinical quantification of injury. Several multi-parametric MRI data sets were acquired such as MPRAGE, DWI, SWI, FLAIR, and ADC. Of these data sets, ADC was deemed to be quantitatively the most discriminatory for the injury types assessed clinically, and utilized for the comparison between computational simulation and clinical MRI. The ADC allowed representing the changes in the state of the tissues due to the insult by parameterizing and representing the degree of diffusion of water molecules to identify multiple forms of brain injury (Sener, 2001; Hagmann et al., 2006; Nelson et al., 2008; Tong et al., 2008). ADC also identified transition of a volume in the brain occupied by CSF (with higher diffusion values due to large water content) to a mixture of blood (with lower diffusion values due to less water content) or other fluids and CSF, and quantified such changes. ADC is limited because it provides an average value over a plurality of layers (i.e., thicker slices of brain) from T1 and T2 MRI slices. This reduces the resolution and sensitivity to an extent in a given plane when compared with the sensitivity in T1 and T2 data (The details in T2 shown in Supplementary Materials apply for T1 data; not shown here).

Computational biomechanical analyses of these cases primarily address the energy distribution and absorption from blunt impact to the head and the brain, and the biomechanical parameters that correlate with clinical imaging of such insults. For blunt impact insults, the energy is transferred from the skull into the brain. Positive (compressive) and negative (tensile) pressure waves as well as shear stress waves could be generated in the brain tissue. In most biological tissues, tensile stress is more injurious than the compression mostly because the interstitial fluid cannot resist the tension in supporting tissue structural components. Compared with pressure waves, shear waves propagate within the brain at a much slower speed, last longer, and cause larger deformations of tissues and tearing of adjacent structures. Strain rate represents how fast the shear is happening with respect to time. For the viscoelastic brain tissue, material shearing behaves differently with the increase in strain rates. Figure 7 showed our consideration of a multitude of biomechanical measures to identify the best mapping parameters with ADC maps and clinical assessment of the injury for both the cases.

Hydrostatic pressure is used here because it includes stresses in multiple directions by considering the mean of normal stress components in the tissue. Gradual increase in pressure, as in deep sea diving, where the pressures are excessive, does not adversely affect a diver while diving deeper. However, it is well known that if the diver comes up to the sea level quickly, this rapid ascent creates a pressure difference between the inside of the body and the outside, and can subject the diver’s organs to tensile loading, which is injurious to the tissues. The same phenomenon is possible in brain tissue due to large negative pressures when the head is subjected to impacts or shock fronts.

For the two cases studied here, strain rate and tensile pressure show correspondence with quantifiable changes in ADC values in the tissue and clinical diagnosis from evaluation of MRI images. It is conceivable that in case 1, with the high strain rate in the coup region (but not in the contrecoup region), the impact yields very localized deformation patterns, as supported by high ADC values in this region. Likewise, in case 2 with the head impacting a larger object at a lower velocity, the momentum distribution is over a larger area showing no localized deformation. In this case, the tensile pressure shows a closer correspondence with the ADC values in both coup and contrecoup regions.

Based on the above correspondence, we develop a methodology that can map clinical diagnosis, clinical information, and quantitative data, with biomechanical simulation data. The key is creating a biomechanical analogy of quantitative measurements from clinical assessment, e.g., ADC, and establishing how these biomechanical measures can be used as mapping or equivalent corresponding parameters. As more clinical cases are collected and quantified, biomechanical simulation data can be generated by model reconstruction of incidents using the approach as presented in this paper. With more cases analyzed, the aggregated knowledge of correlating biomechanics and clinical outcomes will improve the understanding for various injury classifications. Artificial intelligence tools and machine learning algorithm utilizing such knowledge can then be created to predict the nature of the insults and injury modalities of brain (Raj et al., 2019).

Future research can cross-validate these findings using a large dataset to relate biomechanical assessment of TBI with image-based assessment and analysis. Other avenues are to explore possible correspondence between biomechanical prediction and other MRI data such as fractional anisotropy (FA) values representing demyelination due to oligodendrocyte ischemia and subsequent apoptosis, as well as explore injury modalities due to blast overpressure and ballistic impact loadings.

### Assumptions and Limitations

There are several limitations in this study: (1) We recognize the differences in head sizes in our design of experiments (shown in Supplementary Table 1). Although the differences due to head size variations are small, they may affect the simulation results. Morphing the computational head model to reflect the dimensions of the actual head geometry could improve simulation accuracy and mapping with clinical image-based data. Advanced morphing techniques such as the ones developed in Refs. (Jolivet et al., 2015; Zhang et al., 2017; Hu, 2018) could be utilized and extended to develop a subject-specific head model based on the current head model. Incorporating such modeling nuances to quantify errors introduced by using a 50th percentile human head computational model has not been addressed in this paper. (2) We have used simplified material models for different tissues. For example, we modeled the cortical and trabecular skull bones as isotropic elastoplastic materials, as found in the literature, although the skull bone is recognized to be anisotropic in nature and has been known to exhibit viscoelastic strain-rate sensitive behavior under dynamic loading. Another material model aspect that we did not consider is person-to-person variability of human tissue properties. We did not consider such uncertainties treating them as higher-order factors. (3) We treated the CSF in the head as a highly incompressible elastic solid. Treating the CSF as a fluid and applying a fluid–tissue interface to solve the fluid–structure interaction between the CSF and surrounding tissues by a sophisticated coupled fluid–solid solver might refine the approach but would add significant complexity and computational challenge. (4) The accidents in both cases were reconstructed based on information from the hospital where emergency care was provided to the patients, eyewitness reports, clinical assessment reports, and imaging data by the clinicians. Unlike the blunt impact data obtained from a controlled scientific experiment, the reconstruction of uncontrolled real-world incidents such as the two cases may not be unique. We assumed that the impactor is a stone with a spherical shape with a radius that is varied for different cases. Using a non-smooth surface or object with multiple edges will increase the complexity of computational simulation, but may not be consequential to refining the methodology. (5) Due to logistical constraints, we recognize that there is inevitably a time delay between the accident and the MRI scan at the hospital. During this period, the level of interstitial edema may increase slowly following the injury. However, the region of ADC abnormality caused by hemorrhage is unlikely to change significantly within this time frame (Gasparetto et al., 2011). (6) Longer simulation times combined with the proper simulation of large brain rotation can predict other outcomes that may be necessary for lower speed impact loading conditions. In the future, applying the techniques in this paper to other head trauma cases, together with proper pattern match and statistical analyses, will result in more objective correlative metrics, which enhance biomechanical simulations in predicting the injury risk and patterns and can inform additional diagnosis considerations to the attending physicians.

## Conclusion

We showed a methodology to map computational biomechanical simulation results for blunt impact loading with clinical brain imaging data for two cases. Biomechanical parameters such as pressure, shear stress, principal strain, strain rate, product of strain and strain rate, and strain energy were considered to quantitatively compare biomechanical simulations with clinical assessments. Based on the simulation result analysis, we found different biomechanical measures to explain different blunt impact injury modalities. The minimum pressure (i.e., maximum tensile pressure) and maximum strain rate in brain tissue were seen to best represent tissue damage/injury identified by *in vivo* ADC values from MRI analysis. Specifically, for case 1, a blunt impact with a small solid object resulting in an indented skull, the ADC contours of contusion corresponded well with those for effective strain rates higher than 250 s^–1^. For case 2, a blunt impact with a larger solid object producing a coup–contrecoup injury and no noticeable skull indentation, the ADC contours showing contusion and edema mapped well with a negative pressure of -90 kPa or more. The methodology comparing biomechanical simulations with ADC from image-based clinical analysis presented here can lead to a future roadmap to understand and interpret injury criteria, and to improve accuracy in biomechanical prediction.

## Data Availability Statement

The original contributions presented in the study are included in the article/Supplementary Material, further inquiries can be directed to the corresponding author/s.

## Ethics Statement

The studies involving human participants were reviewed and approved by the written, informed consent was obtained from the individuals for the publication of any potentially identifiable images or data included in this article. The institutional Ethics Committee for Institute of Nuclear Medicine and Allied Sciences (INMAS) approved the study. The patients/participants provided their written informed consent to participate in this study.

## Author Contributions

Methodology and data processing were conceptualized by XT, VS, and AB. XT conducted the modeling and simulation and led the data analysis and documentation. VS analyzed the imaging data, contributed to the data analysis and documentation. MD’S collected the imaging and patient case history data, analyzed the clinical data, and contributed to the documentation. RG, JL, and AS helped with the technical discussions and technical editing. AB contributed to the data analysis, documentation, discussions, and interfacing between the four organizations contributing to this manuscript. All authors contributed to the article and approved the submitted version.

## Conflict of Interest

The authors declare that the research was conducted in the absence of any commercial or financial relationships that could be construed as a potential conflict of interest.

## Supplementary Material

Supplementary Figure 1 Case 1 of skull indentation: medical images (FLAIR, CSF in dark) on axial/transverse planes.

Supplementary Figure 2 Case 2 of coup-contrecoup injury: medical images (FLAIR, CSF in dark) on axial/transverse planes.

Supplementary Figure 3 Contours of different strain rate measures showing similar patterns but with different magnitudes.

Supplementary Figure 4 Correspondence between ADC values and maximum effective strain rate at coup location in transverse plane of case 1. Values are plotted along horizontal line through the coup between points C and A. 1, 2, and 3 mark the location filled with fluid, C stands for coup region. The dotted line represents approximate mean ADC value of brain tissue.

Supplementary Figure 5 Correspondence between ADC values and minimum pressure at showing coup location in transverse plane at the coup of case 2. Values are plotted along for the line through the contrecoup between points A and CC. 1, 2, and 3 mark the location filled with fluid, CC stands for contrecoup regions. The dotted line represents approximate mean ADC value of brain tissue.

Supplementary Table 1 Comparison of heads of case 1 and case 2 with computational head model.
