# Supplementary figures and images for: A Methodology to Compare Biomechanical Simulations With Clinical Brain Imaging Analysis Utilizing Two Blunt Impact Cases
Source: Front Bioeng Biotechnol. 2021 Jul 1;9:654677. doi: 10.3389/fbioe.2021.654677 (PMC8280347; doi:10.3389/fbioe.2021.654677)

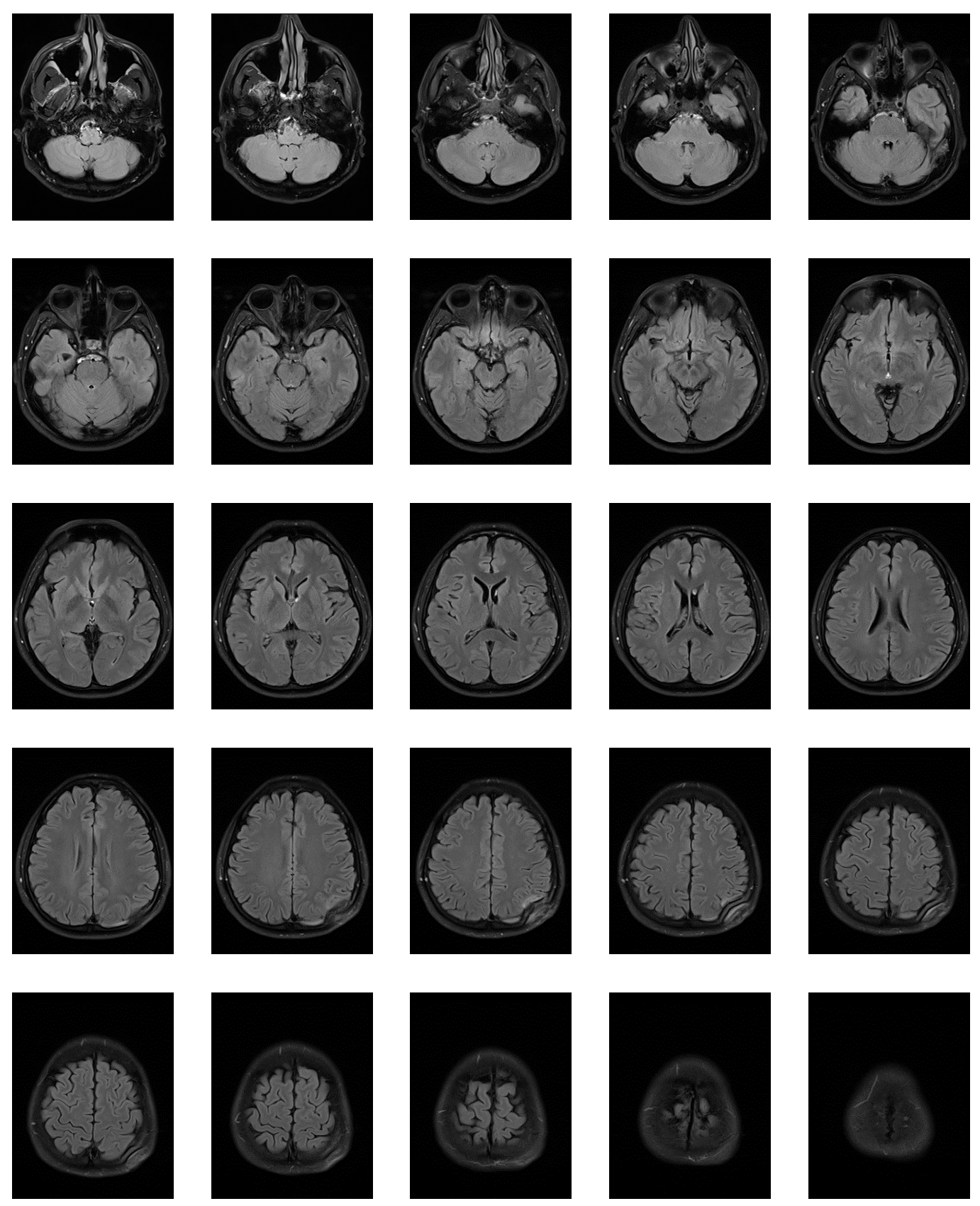

Supplement: Supplementary Figure 1 — Case 1 of skull indentation: medical images (FLAIR, CSF in dark) on axial/transverse planes. [file Image_1.tif]

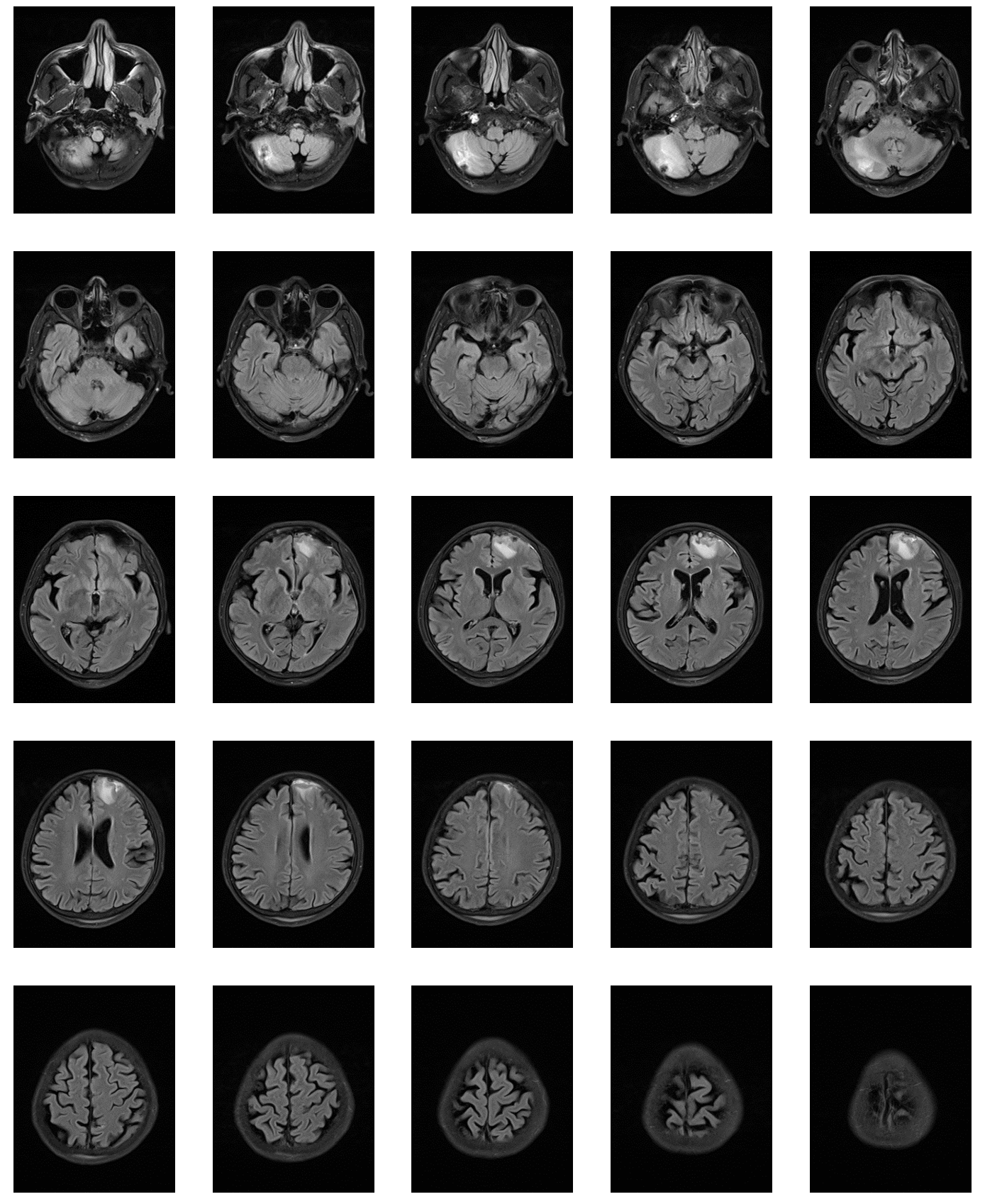

Supplement: Supplementary Figure 2 — Case 2 of coup-contrecoup injury: medical images (FLAIR, CSF in dark) on axial/transverse planes. [file Image_2.tif]

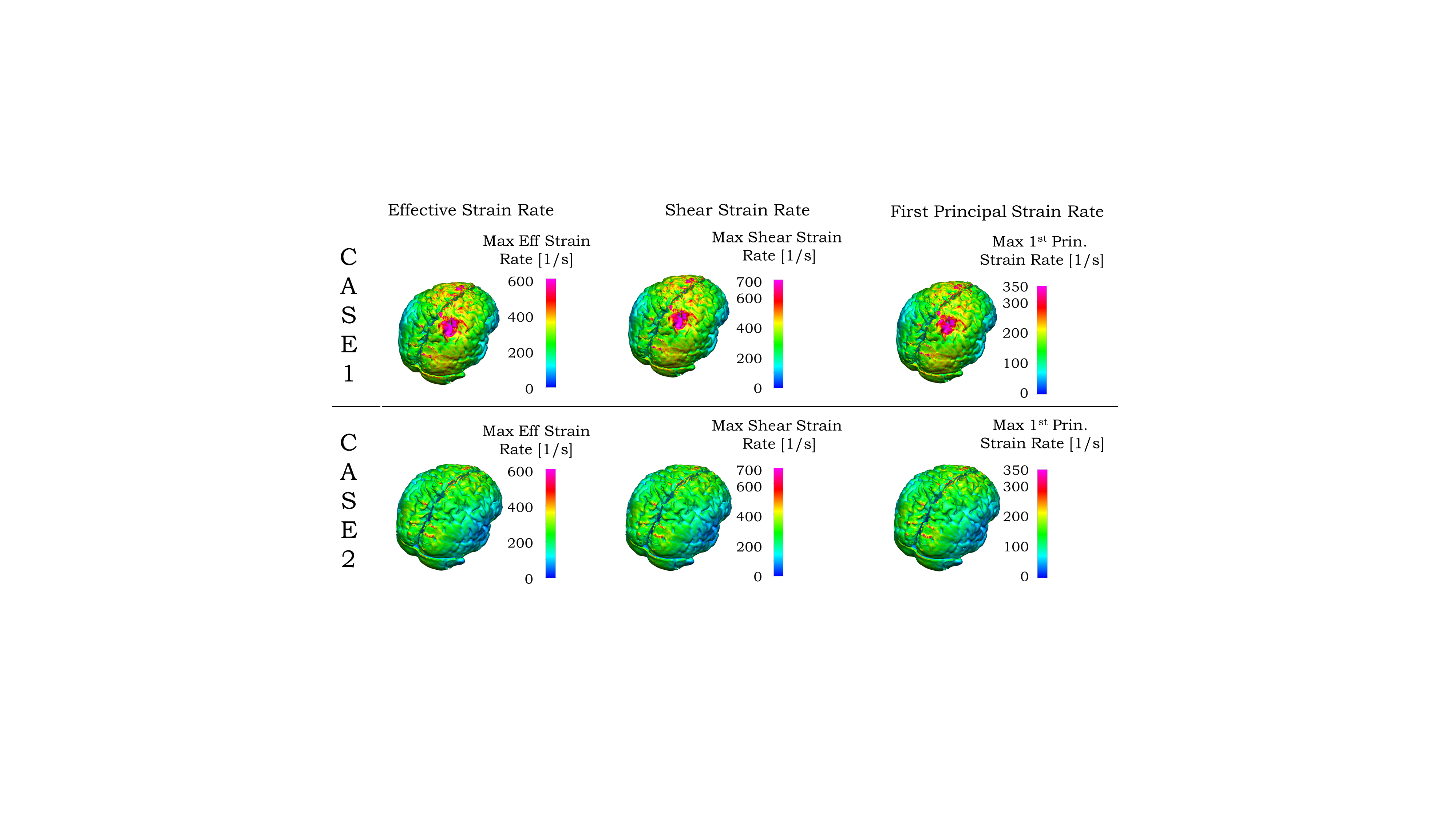

Supplement: Supplementary Figure 3 — Contours of different strain rate measures showing similar patterns but with different magnitudes. [file Image_3.tif]

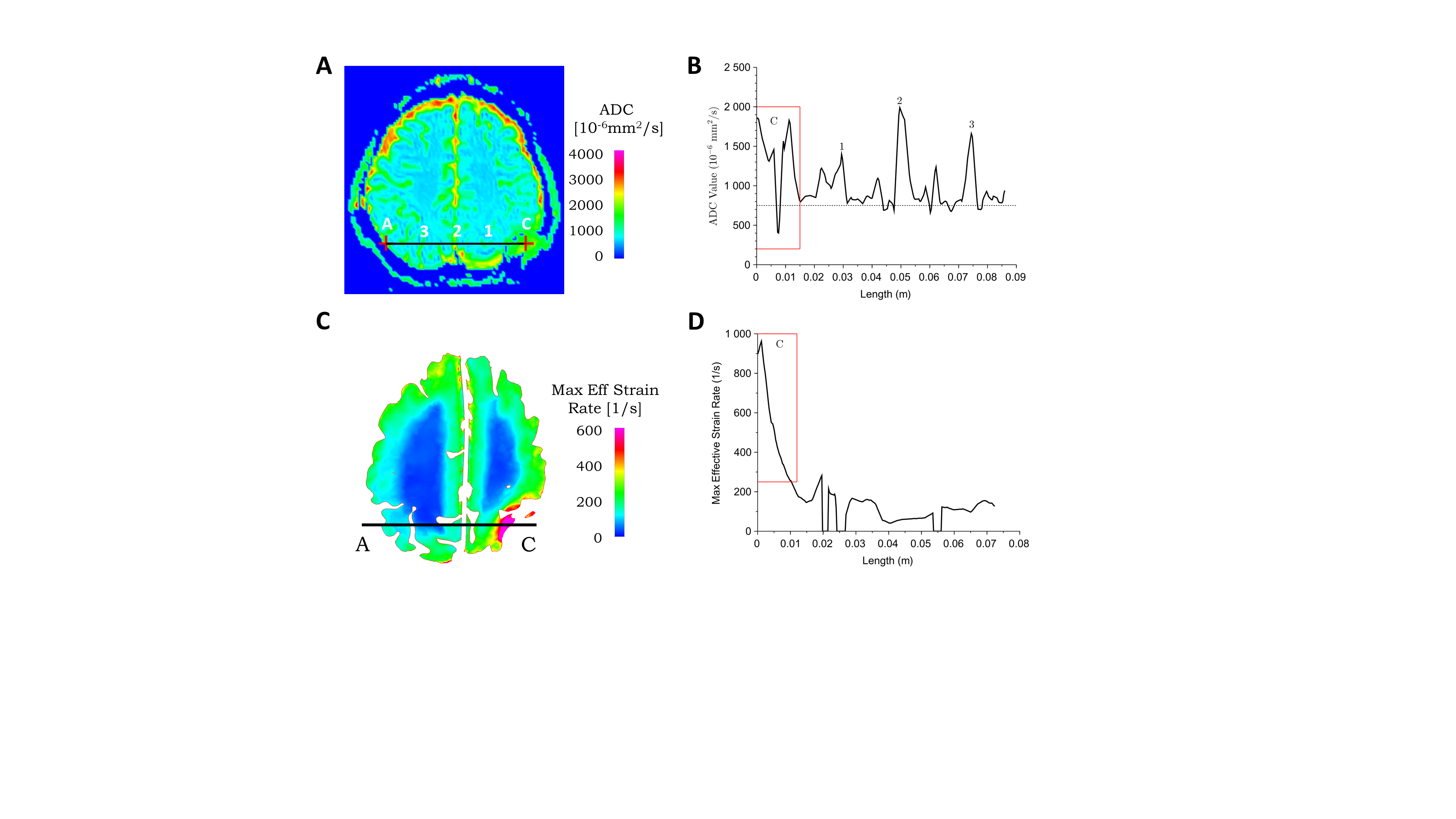

Supplement: Supplementary Figure 4 — Correspondence between ADC values and maximum effective strain rate at coup location in transverse plane of case 1. Values are plotted along horizontal line through the coup between points C and A. 1, 2, and 3 mark the location filled with fluid, C stands for coup region. The dotted line represents approximate mean ADC value of brain tissue. [file Image_4.tif]

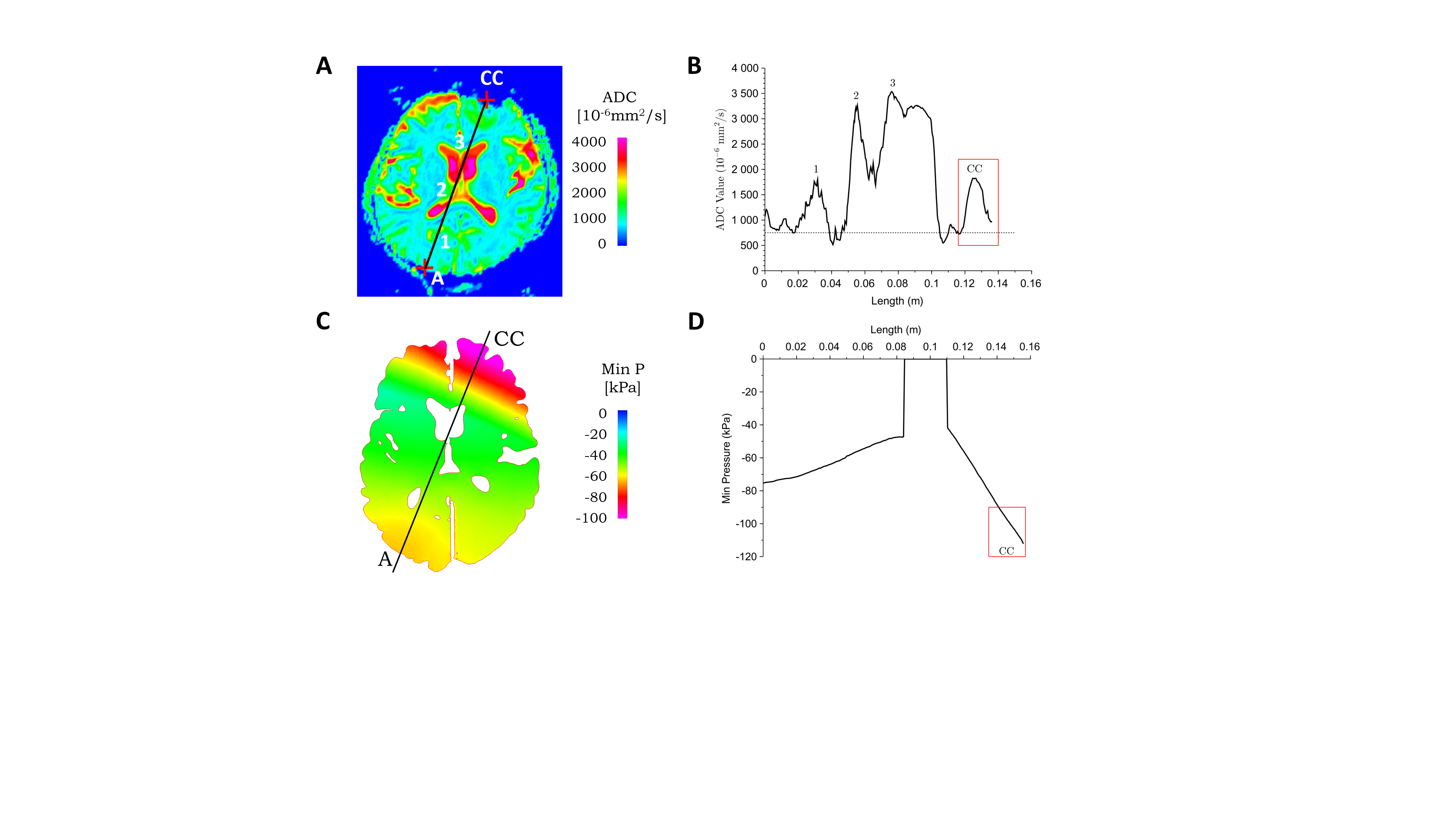

Supplement: Supplementary Figure 5 — Correspondence between ADC values and minimum pressure at showing coup location in transverse plane at the coup of case 2. Values are plotted along for the line through the contrecoup between points A and CC. 1, 2, and 3 mark the location filled with fluid, CC stands for contrecoup regions. The dotted line represents approximate mean ADC value of brain tissue. [file Image_5.tif]
