# Supplementary material for: A Methodology to Compare Biomechanical Simulations With Clinical Brain Imaging Analysis Utilizing Two Blunt Impact Cases
Source: Front Bioeng Biotechnol. 2021 Jul 1;9:654677. doi: 10.3389/fbioe.2021.654677 (PMC8280347; doi:10.3389/fbioe.2021.654677)
Supplement: Supplementary Table 1 — Comparison of heads of case 1 and case 2 with computational head model. [file Table_1.pdf]

Table S1. Comparison of heads of case 1 and case 2 with computational head model.

	Case 1	Case 2	Head Model
Anterior-Posterior (Sagittal Plane)	191 mm	180 mm	191 mm
Side-Side (Transverse plane)	170 mm	158 mm	160 mm
Top-Foramen Magnum (Sagittal Plane)	162 mm	153 mm	153 mm
